# Supplementary material for: Development of a computer-aided design software for the quantitative evaluation of aesthetic damage
Source: PLoS One. 2019 Dec 18;14(12):e0226322. doi: 10.1371/journal.pone.0226322 (PMC6919621; doi:10.1371/journal.pone.0226322)
Supplement: S1 Code site — 1. (ZIP) [file pone.0226322.s001.zip › [EESC_jr][Projeto]Relatorio_07_21/reports/templates_zip/gentelella-master/vendors/morris.js/examples/bar-colors.html]

# Bar charts

```
// Use Morris.Bar
Morris.Bar({
  element: 'graph',
  data: [
    {x: '2011 Q1', y: 0},
    {x: '2011 Q2', y: 1},
    {x: '2011 Q3', y: 2},
    {x: '2011 Q4', y: 3},
    {x: '2012 Q1', y: 4},
    {x: '2012 Q2', y: 5},
    {x: '2012 Q3', y: 6},
    {x: '2012 Q4', y: 7},
    {x: '2013 Q1', y: 8}
  ],
  xkey: 'x',
  ykeys: ['y'],
  labels: ['Y'],
  barColors: function (row, series, type) {
    if (type === 'bar') {
      var red = Math.ceil(255 * row.y / this.ymax);
      return 'rgb(' + red + ',0,0)';
    }
    else {
      return '#000';
    }
  }
});
```
